# Osteomyelitis of the jaws in patients with pycnodysostosis: a systematic review

**DOI:** 10.1016/j.bjorl.2020.12.009

**Published:** 2021-01-29

**Authors:** Glória Maria de França, Fernanda Aragão Felix, Everton Freitas de Morais, Maurília Raquel de Souto Medeiros, Ana Cláudia de Macedo Andrade, Hébel Cavalcanti Galvão

**Affiliations:** a Universidade Federal do Rio Grande do Norte (UFRN), Departamento de Odontologia, Programa de Pós-Graduação em Ciências Odontológicas, Área de Concentração em Patologia Oral e Estomatologia, Natal, RN, Brazil; b Universidade Federal do Rio Grande do Norte (UFRN), Centro de Biociências, Programa de Pós-Graduação em Ciências Odontológicas, Área de Concentração em Biologia Oral, Natal, RN, Brazil

**Keywords:** Pycnodysostosis, Cathepsin K, Osteomyelitis, Diagnosis, Jaws

## Abstract

**Introduction:**

Pycnodysostosis is a rare autosomal recessive syndrome that provides the abnormal bone metabolism that increases the susceptibility of patients to develop osteomyelitis.

**Objective:**

This systematic review was conducted to analyze the risk factors associated with the development of complications in the jaws (fractures and osteomyelitis), as well as their clinical-pathological characteristics and therapeutic approaches in patients with pycnodysostosis.

**Methods:**

Searches were performed in the PubMed, Web of Science, Scopus, Lilacs, and Cochrane databases. Case reports or case series that met the eligibility criteria according to the PRISMA statement were included. The full texts of 31 articles were retrieved. Twenty of these articles published between 1969 and 2018 were selected, which described 26 cases of osteomyelitis in patients with pycnodysostosis.

**Results:**

The mean age of the patients was 37.84 years; the male-to-female was 1.36:1. The mandible was the most affected site (76.9%). Tooth extraction was the main risk factor for osteomyelitis (61.5%), followed by infection (26.8%) and mandibular fracture (23.0%). Antibiotic therapy alone or combined with some surgical procedure was the treatment used in most cases (80.7%).

**Conclusion:**

The findings of this review showed that patients with pycnodysostosis are more likely to develop osteomyelitis of the jaws after surgical procedures, especially tooth extraction which remains the main risk factor for its establishment. In addition, prophylactic antibiotic-therapy in the pre- and postoperative periods may prevent the development of osteomyelitis in pycnodysostosis.

## Introduction

Pycnodysostosis (PYCD) is a rare autosomal recessive syndrome that belongs to the group of bone dysplasias. This disorder is caused by mutations in the CTSK gene located on chromosome 1q21^1^, ^2^ that encodes cathepsin K, a lysosomal cysteine protease present in osteoclasts which participates in the degradation of type I collagen (predominant protein in the bone matrix). Mutations in this gene lead to defective bone metabolism, more specifically deficient bone resorption.^2^, ^3^, ^4^

The prevalence of PYCD is 1 in 1.7 million and no sex predilection has been reported.^1^ Autosomal recessive inheritance is the pattern of transmission of mutations in the CTSK gene that encodes cathepsin K. Thus, parental consanguinity is an important etiological factor.^3^, ^4^, ^5^ PYCD is characterized by abnormal bone metabolism, reduced bone elasticity, endocrine and immunological dysfunction and altered extracellular matrix remodeling in organs such as the lung, thyroid and skin.^1^, ^3^, ^5^

Patients with PYCD commonly exhibit a short-stature phenotype, open bone sutures, short phalanges, bone dysplasia with altered density of the bone matrix, and pathological fractures as a result of reduced elasticity and poor bone healing. Oral manifestations include an obtuse mandibular angle, malocclusion, an ogival palate, and dental anomalies.^1^, ^6^ The abnormal bone metabolism increases the susceptibility of patients to develop osteomyelitis. In gnathic bones, local factors related to the development of osteomyelitis include the bone characteristics of the maxilla and mandible, continuous traumas, alveolar surgical procedures, and conditions that affect the teeth such as periapical and periodontal disease.^7^, ^8^, ^9^ It should be emphasized that the treatment of osteomyelitis in gnathic bones is difficult and may require major surgical resections.^5^

Therefore, the hypothesis of this study is that manipulation of the jaws in patients with PYCD may favor the development of osteomyelitis. This systematic review was conducted to analyze the risk factors associated with the development of complications in the jaws (fractures and osteomyelitis), as well as their clinical-pathological characteristics and therapeutic approaches in patients with PYCD.

## Methods

A systematic literature review on PYCD based on the 2009 PRISMA (Preferred reporting items for systematic reviews and meta-analyses) statement^10^ was conducted on 6 March 2019 to answer the following research question according to the PICO criterion (population, intervention, control, and outcome): “Is surgical manipulation of the jawbones the main risk factor for osteomyelitis in patients with PYCD?”. This systematic literature review was registered in PROSPERO (International prospective register of systematic reviews) under protocol number: CRD42020209580.

The databases selected for the systematic review were PubMed, Web of Science, SCOPUS, Cochrane, and LILACS. The following search terms were used: “Pycnodysostosis” OR “Pyknodysostosis” AND “Surgery, Oral” OR “Maxillofacial Surgery” OR “Oral Surgery” OR “Exodontics” OR “Dentistry, Operative” OR “Surgical Procedures, Oral” OR “Procedure, Oral Surgical” OR “Procedures, Oral Surgical” OR “Surgical Procedure, Oral” OR “Maxillofacial Procedures” OR “Maxillofacial Procedure” OR “Procedure, Maxillofacial” OR “Procedures, Maxillofacial” OR “Tooth extraction” AND “Osteomyelitis” OR “Osteomyelitides” OR “Diseases, Infectious Bone” OR “Bone Diseases, Infectious” OR “Diseases, Infectious Bone” OR “Infectious Bone Disease” OR “Infectious Bone Diseases” (Supplementary Table 1).

The criteria for inclusion were published studies specifically investigating osteomyelitis of the jaws in patients with PYCD. Only case reports and case series were included. Excluded were studies that did not address osteomyelitis as a complication in the jaws, studies addressing osteomyelitis in bones other than the jaws, articles that did not report the primary cause of osteomyelitis, studies based on animal models, *in vitro* studies, editorial letters, review articles, and studies whose full text was not available through inter-library loan.

The articles were selected independently by two of the authors. First, the authors selected articles by reading the title and abstract, according to the established inclusion and exclusion criteria. Articles not related to the topic were excluded. The full text of the remaining studies was downloaded, and the articles were selected based on the eligibility criteria. Articles whose full text was not available were excluded and the final sample of studies for qualitative synthesis was determined. Discrepancies were resolved by a third author.

The eligible studies were reviewed and the following data were extracted: (1) authors and year of publication; (2) number of patients; (3) sex and age (mean) of the patient; (4) cause; (5) clinical (family history, location, symptoms) and radiographic characteristics; (6) therapeutic intervention and outcome, (7) and patient follow-up. The reviewers did not disagree on the selection of the articles. The methodological quality of the articles was evaluated according to the CARE guidelines for case reports, a qualitative checklist for observational studies and case reports.

## Results

### Overall results

**Figure 1:**
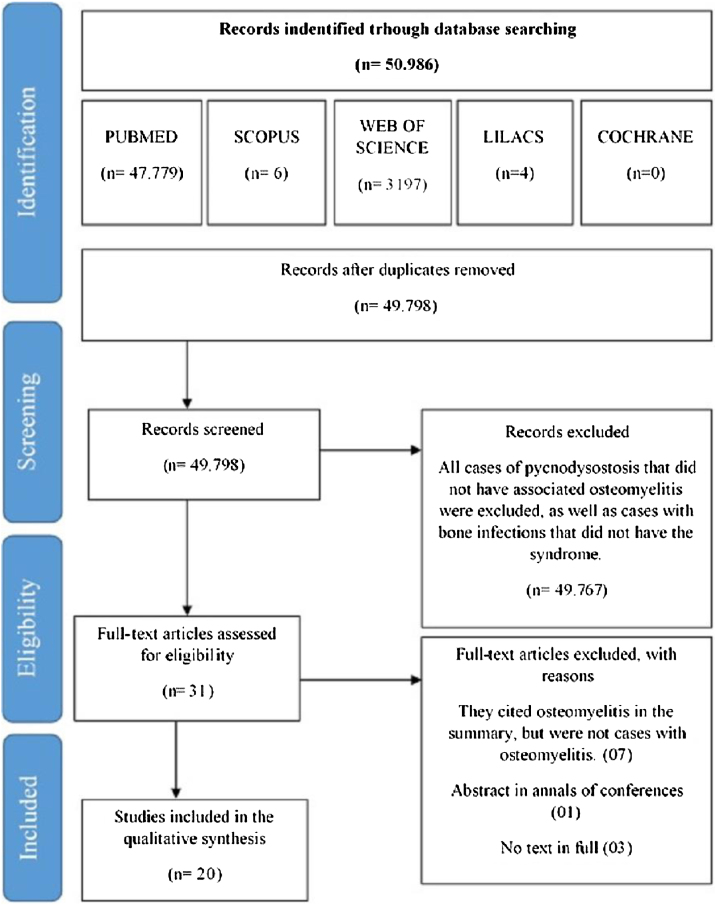
Flow chart of the database search and article selection process according to PRISMA.

The electronic database search was last updated in March 2019 and yielded 50,986 records: 47,779 from PubMed, 3197 from Web of Science, 4 from LILACS, 6 from SCOPUS, and 0 from The Cochrane Library. A total of 49,798 records were obtained after the removal of duplicates, which were screened according to the inclusion and exclusion criteria. At this stage, 31 articles were considered relevant to the topic (the other 49,767 studies did not specifically address PYCD). The full texts of these 31 articles were then assessed for eligibility, leading to the inclusion of 20 studies. The flow chart of the study selection process adapted from the PRISMA statement is shown in Fig. 1. Finally, 20 articles published between 1969 and 2018 were retrieved by the authors, corresponding to 26 cases of osteomyelitis in PYCD. The etiological, clinical, histological, biological, and therapeutic data for each of the 26 patients included in the study were analyzed. Due to the heterogeneity of the available literature and the lack of data in several studies, the authors decided to limit themselves to a descriptive statistical analysis and not to perform a meta-analysis.

### Quality assessment

The quality of the articles was established using the CARE guidelines for case reports (2013) and the results are shown in Table 1. Of the 29 items of the CARE checklist (2013), the study of Kirita et al.^11^ adhered to the largest number of items (20 items), followed by the studies of Kamat et al.,^2^ Fénelon et al.^12^ (18 items), Kato et al.,^13^ and Frota et al.^14^ (17 items). In contrast, the case reports by Emami-Ahari et al.^15^ and Dimitrakopoulos et al.^16^ adhered to only few items (6 and 8 items, respectively). The clinical findings topic was the most frequently reported item, which was described in all articles (n = 20). On the other hand, the prognosis item was not addressed by any of the studies. The item “diagnostic reasoning including other diagnoses considered” was only reported in the study of Dhameliya et al.^17^

**Table 1:** Quality assessment to CARE checklist.

Topic	Item	Description	Percentage of case reports
Title	1	The words “case report” should be in the title along with the area of focus	7
Keywords	2	2 to 5 keywords that identify areas covered in this case report	10
Abstract	3a	Introduction – What is unique about this case? What does it add to the medical literature?	5
	3b	The main symptoms of the patient and the important clinical findings	6
	3c	The main diagnoses, therapeutics interventions, and outcomes	6
	3d	Conclusion – What are the main “take-away” lessons from this case?	5
Introduction	4	One or two paragraphs summarizing why this case is unique with references.	17
Patient information	5a	De-identified demographic information and other patient specific information.	13
	5b	Main concerns and symptoms of the patient.	16
	5c	Medical, family, and psychosocial history including relevant genetic information (also see timeline)	15
	5d	Relevant past interventions and their outcomes	13
Clinical findings	6	Describe the relevant Physical Examination (PE) and other significant clinical findings.	20
Timeline	7	Important information from the patient’s history organized as a timeline	16
Diagnostic assessment	8a	Diagnostic methods (such as PE, laboratory testing, imaging, surveys)	17
	8b	Diagnostic challenges (such as access, financial, or cultural)	1
	8c	Diagnostic reasoning including other diagnoses considered	1
	8d	Prognostic characteristics (such as staging in oncology) where applicable	0
Therapeutic intervention	9a	Types of intervention (such as pharmacologic, surgical, preventive, self-care)	18
	9b	Administration of intervention (such as dosage, strength, duration)	10
	9c	Changes in intervention (with rationale)	2
Follow-up and outcomes	10a	Clinician and patient-assessed outcomes (when appropriate)	13
	10b	Important follow-up diagnostic and other test results	9
	10c	Intervention adherence and tolerability (How was this assessed?)	4
	10d	Adverse and unanticipated events	4
Discussion	11a	Discussion of the strengths and limitations in your approach to this case	4
	11b	Discussion of the relevant medical literature	13
	11c	The rationale for conclusions (including assessment of possible causes)	13
	11d	The primary “take-away” lessons of this case report	3
Patient perspective	12	When appropriate the patient should share their perspective on the treatments, they received	3

### Epidemiology and causal factors

Twenty-six cases of osteomyelitis of the jaws related to PYCD were described in the 20 selected articles. Men were more commonly affected (n = 15) than women (n = 11), with a male-to-female ratio of 1.36:1. The age of the patients ranged from 21 to 55 years (mean of 37.84 ± 9.58 years), as described in Table 2.

**Table 2:** Etiology, Clinical and radiographic characteristics of the 27 cases of osteomyelites in patients with pycnodysostosis.

Parameters	n	%
Nº de casos	26	100.0
Age		
Range	21–55 years old	
Media	37.84	
Standard deviation	9.58	
Gender		
Female	11	42.3
Male	15	57.7
Family history^a^		
Consanguineous marriage	4	15.4
Siblings	6	23.0
Aunt	1	3.8
No family history	6	23.0
NA	11	42.3
Medical history^a^		
Fractures of the long bones	9	34.6
Femoral fracture	4	15.4
Tibial fractures	3	11.5
Elbows	3	11.5
Rib	2	7.7
Hip	2	7.7
Clavicle	1	3.8
Shoulder blade	1	3.8
Knee	1	3.8
Hydrocephalus	2	7.7
NA	7	26.9
Promoter factor^a^		
Tooth exodontics	16	61.5
Jaw fracture	6	23.0
Carie	3	11.5
Periodontal disease	3	11.5
Carie and periodontal disease	1	3.8
Anatomical location		
Maxila	4	15.4
Mandíbula	20	76.9
Maxila e mandíbula	2	7.7
Symptomatology^a^		
Swelling	12	46.1
Pain	11	42.3
Purulent secretion	9	34.6
Fistula	5	19.2
Trismus	1	3.8
No symptomatology	1	3.8
NA	2	7.7
Radiographic features^a^		
Sequestrum	16	61.5
Osteolysis	7	26.9
Radiolucence	2	7.7
Poorly defined edges	2	7.7
Osteosclerosis	11	42.3
Jaw fracture	4	15.4
Hipercementosis	3	11.5
Treatment^a^		
Antibiotic therapy	21	80.7
Sequestrectomy	14	53.8
Curettage	8	30.7
Tooth extraction	4	15.4
Resection	2	7.7
Jaw reconstruction	5	19.2
NA	1	3.8
Follow-up		
Present in	17	65.4
Range	6–168 months	
Media	28.46	
Standard deviation	39.80	
NA	9	34.6

NA, Not available.

a Patients present more than one category in the group.

The family history of PYCD was not reported in eight articles, corresponding to 11 cases without information. Most of the articles in which this information was available reported consanguinity or a family history of the disease: siblings (n = 6), children of consanguineous parents, especially cousins (n = 4) and paternal aunt (n = 1). Six patients had no family history of the disease.

The medical history revealed a history of some type of bone fracture, especially in the long bones (n = 9). The bone fractures involved the femur (n = 4), tibia (n = 3), elbows (n = 3), ribs (n = 2), hip (n = 2), clavicle (n = 1), scapula (n = 1), and knee (n = 1). Two patients had a history of hydrocephalus.

**Figure 2:**
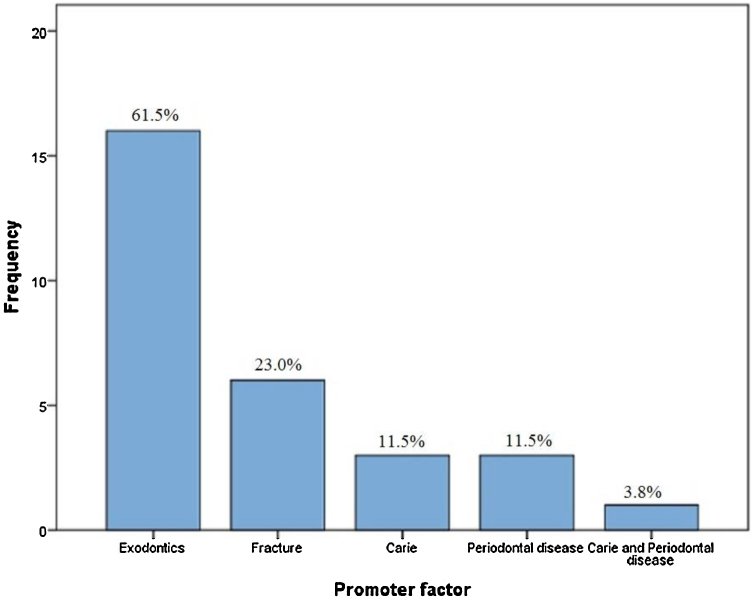
Causes of osteomyelitis in patients with pycnodysostosis.

**Figure 3:**
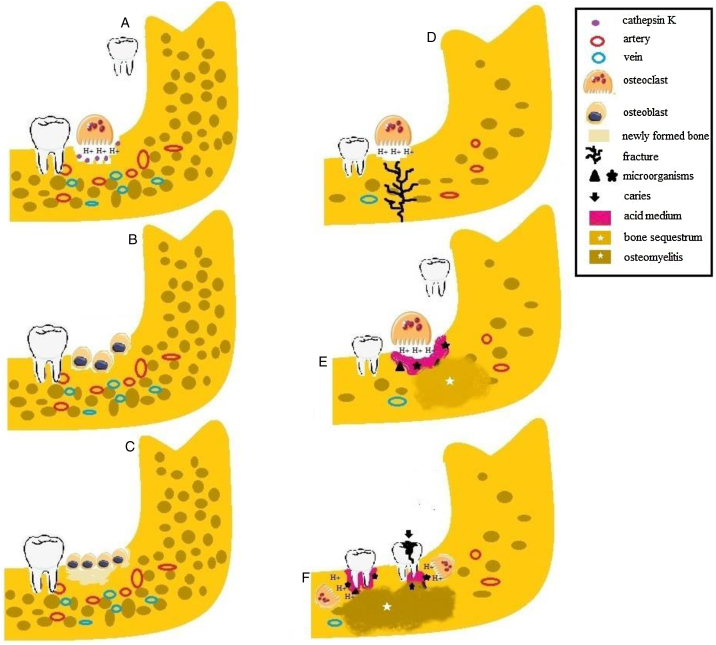
Etiology of osteomyelitis in patients with pycnodysostosis. (A) Osteoclasts release H + and cathepsin K into How ship lacunae during tooth extraction in patients with normal bone density. (B) After bone resorption, osteoblasts are attracted to the site and initiate the process of bone neoformation. (C) Newly formed bone. (D) In the absence of cathepsin K, osteoclasts act inefficiently, leading to acid medium. Patients with osteosclerosis are more susceptible to fractures in response to small traumas because of reduced bone vascularization. (E) The presence of microorganisms, the acid medium and the impairment of bone turnover cause the formation of bone sequestrum. (F) Caries and periodontal disease induce the release of inflammatory mediators and microbial products and associated with impaired osteoclast activity, promote osteomyelitis in patients with pycnodysostosis.

Among the 26 cases of osteomyelitis related to PYCD found in the literature, tooth extraction was the main risk factor in 16 cases (61.5%), followed by a history of jaw fracture (n = 6, 23.0%) and the presence of infection such as caries (n = 3, 11.5%) and periodontal diseases (n = 3, 11.5%). The simultaneous presence of caries and periodontal lesions was observed in one case (3.8%), as shown in Fig. 2. All risk factors for osteomyelitis development are illustrated in Fig. 3.

### Clinical and radiographic characteristics

Regarding the anatomical location, there was a slight predominance of osteomyelitis in the mandible (n = 20, 76.9%) over the maxilla (n = 4, 15.4%). Both jaws were affected in two cases (7.7%). The most common symptoms were swelling and edema (n = 12), followed by pain (n = 11), purulent discharge (n = 9), presence of a fistula (n = 5), and trismus (n = 1). Information about symptoms was not available in two cases.

As radiographic characteristics, osteomyelitis was accompanied by bone sequestrum (n = 16), osteolysis (n = 7), radiolucency (n = 2), and ill-defined borders (n = 2). Increased bone density (osteosclerosis) was observed in 11 of the cases and was the most common pattern after bone sequestrum. In four cases, fracture of the jaws was demonstrated by radiography. Hypercementosis involving the permanent teeth was seen in three cases.

### Therapeutic interventions and followup

The treatment of osteomyelitis ranged from conservative (antibiotic therapy and curettage) to more radical approaches (tooth extraction, sequestrectomy, and resection). Antibiotic therapy was the treatment of choice in 21 cases, followed by sequestrectomy (n = 14), curettage (n = 8), tooth extraction (n = 4), and resection (n = 2).

Of the 21 cases receiving antibiotic therapy, thirteen of them specified the antibiotic used, although only eight reported the dosage and time used (Supplementary Table 2). Culture and antibiogram were performed in 3 articles, including carbenicillin and ampicillin,^18^ clindamycin,^14^ moxifloxacin (400 mg once daily), injection cefotaxime sodium (1 g twice daily), and injection metronidazole (500 mg thrice daily).^2^ Within the penicillin group, the use of penicillin G with an average concentration of 11.2 IU was selected in four of the cases.^16^, ^19^, ^20^ Ampicillin with 500 mg regimen, 3× daily for 5 days, was used in one case;^20^ in another case,^18^ the authors also used ampicillin, but did not specify the adopted regimen. The use of amoxicillin 1 g, 2× daily for 15 days, was adopted in two cases.^12^ In addition, flucloxacillin and ticarcilin-clavulanato were used in one case each, although the regimen was not specified.^21^, ^22^ In the lincosamide class, clindamycin 300 mg was the drug of choice, with a 4× daily regimen for 14 days in one case,^6^ although in the other two articles the regimen was not specified;^14^, ^22^ in addition to lincomycin 500 mg for 10 days, used in one case.^23^

In five cases, the treatment of choice was accompanied by reconstruction of the jawbones. In some cases, prophylactic antibiotic therapy was administered in combination with curettage, extraction, sequestrectomy and resection.^14^ The time of followup was reported in 17 cases and ranged from 6 to 168 months, with a mean of 28.46 ± 39.8 months.

## Discussion

The osteoclasts of patients with PYCD are unable to degrade collagen I and other non-collagenous proteins that form the bone matrix, while these cells maintain their demineralizing activity, resulting in abnormal bone metabolism.^24^, ^25^ The absence of cathepsin K also seems to stimulate cortical bone formation through the negative control of periostin secreted by osteoblasts and osteocytes, a fact that explains the apparent contradictory effect of the increased bone density in PYCD,^26^, ^27^ demonstrated by the presence of osteosclerosis. The latter was observed in 11/26 patients.^12^, ^14^, ^15^, ^17^^,^^20^, ^21^, ^22^, ^23^, ^27^, ^28^, ^29^

The bone quality in patients with PYCD is low (fragile and poorly organized matrix). Bone strength is reduced due to acidification, increasing the risk of fractures, particularly in the lower extremities^1^, ^27^ (Fig. 3D). The present study demonstrated fractures in the long bones of 9/26 patients, corroborating the findings of enhanced mechanical sensitivity and an increased fracture risk in patients with PYCD. These can be explained by the fact that cathepsin K is important for bone resorption in bones with a high turnover rate (e.g., long bones). The present study demonstrated osteolytic lesions in 7/26 patients.^11^, ^13^, ^14^, ^16^^,^^19^, ^22^

The manifestation of osteomyelitis depends on the virulence factors of microorganisms, immune system and vascular factors of the patient. Increased susceptibility to osteomyelitis of the mandible is attributed to the increased bone density (osteosclerosis) favors the establishment of osteomyelitis, which has fewer medullary spaces and a single neurovascular bundle.^17^ Our results were consistent with the literature, with the mandible being the most affected site (n = 22).^2^, ^5^, ^6^, ^11^, ^12^, ^13^, ^14^, ^15^, ^17^, ^18^, ^19^, ^20^, ^21^, ^22^, ^23^, ^27^, ^28^, ^29^, ^30^

In the maxillary bones, osteomyelitis is commonly caused by the dissemination of an odontogenic infection (n = 7)^5^, ^11^, ^12^, ^16^^,^^17^, ^29^ or by surgical traumas (n = 16)^2^, ^5^, ^6^, ^13^, ^14^, ^15^, ^18^, ^19^, ^20^, ^21^, ^22^, ^23^, ^27^, ^28^, ^30^ Since the oral cavity harbors commensal microorganisms, diseases and manipulation of the teeth can create a route for microorganisms to enter the bone.^8^, ^31^ Once it has reached the bone, the inflammatory response may culminate in the production of pus, increasing the intramedullary pressure and reducing blood flow into the bones. An increase in ischemic and necrotic bone contributes to the establishment of infection,^8^, ^32^ demonstrated by the presence of bone sequestrum. The presence of pus can even compromise the integrity of cortical bone. The involvement of adjacent soft tissues is often observed, which is indicated by local edema (12/26)^5^, ^11^, ^13^, ^14^^,^^16^, ^17^, ^19^, ^20^^,^^23^, ^29^ and by intra- or extraoral fistulas,^8^ as found in 5/26 of the reported cases.^14^, ^15^, ^19^, ^23^ Purulent secretion was a finding in 9/26 cases.^2^, ^5^, ^11^, ^15^^,^^17^, ^18^, ^21^ Pain (11/26)^2^, ^6^, ^12^, ^16^^,^^19^, ^20^, ^22^, ^28^^,^^29^ and trismus (1/26),^20^ characteristic of chronic osteomyelitis, the most common type observed in the present study, were also reported.

This study demonstrated that tooth extraction was the main risk factor of osteomyelitis. Such procedures are known to break down the bone barrier and to create a portal of entry for microorganisms that then populate the bone component. In addition, tooth extraction is a traumatic event that, in conjunction with the defective bone matrix in PYCD, favors the occurrence of bone fractures (Fig. 3E). Radiography revealed fractures in four cases.^13^, ^15^, ^17^, ^21^

Osteomyelitis was reported in association with periodontitis in 4/26 cases.^11^, ^16^, ^29^ Periodontitis is a common oral inflammatory disease.^33^ There is evidence indicating that the inhibition of cathepsin K results in the suppression of immune system cells and in the reduction of osteoclast activity, reducing the malnutrition of alveolar bone in periodontitis^34^ (Fig. 3F). Periodontitis may occur in patients with PYCD and can be the primary cause of osteomyelitis, as highlighted in the present study.

Investigation of the role of cathepsin K in periapical diseases demonstrates that this protease is involved in bone resorption and immune system activation, as well as in events during establishment of the immune response related to the recognition of microorganisms by Toll-Like Receptors (TLR) on dendritic cells.^33^ In PYCD, patients therefore lack mechanisms related to the limitation of infection (an effective immune response), a fact favoring the invasion of medullary tissues and the development of osteomyelitis, which was reported in association with pulp events in 4/26 cases.^5^, ^12^, ^16^, ^17^

Antimicrobial therapy and surgical debridement are the main treatment modalities for osteomyelitis.^8^, ^35^ Despite advances in surgical procedures and chemotherapy, osteomyelitis remains difficult to treat and there is no universally accepted treatment protocol.^8^ In the cases of this systematic review, antibiotic therapy was the treatment of choice in patients with osteomyelitis associated with PYCD. This treatment was administered alone in three articles,^2^, ^12^, ^27^ or combined with some surgical procedure in 15 articles.^5^, ^6^, ^11^, ^12^^,^^14^, ^15^, ^16^, ^18^, ^19^, ^20^, ^21^, ^22^, ^23^, ^29^, ^30^ The antibiotic and treatment regime used were specified in most cases.

Culture and antibiogram are laboratory support mechanisms used to reliably determine the causative agent of infection and the most efficient antibiotic therapy to combat it. This procedure was performed in three of the cases of this review.^2^, ^14^, ^18^ In most articles, the antibiotic regimen was combined with the use of two or more medications, including different administration routes (intramuscular, oral, intravenous and topical).^16^, ^18^, ^19^, ^20^, ^21^ Penicillin was the antibiotic of choice in most cases, although in one of the reported cases the antibiogram determined resistance to this antibiotic.^2^ In cultured cases, the antibiotic of choice was variable, including penicillin (carbenicillin and ampicillin), lincosamides (clindamycin), fluoroquinolones (moxifloxacin), cephalosporins (cefotaxime) and nitroimidazole (metronidazole) antibiotics.^2^, ^14^, ^18^

It is argued that radical treatments should be avoided in the management of osteomyelitis associated with PYCD because of the risk of trans- and postoperative complications. In four cases,^2^, ^5^, ^16^, ^17^ the patients refused the surgical approach proposed and chose more conservative treatments. Four of the patients undergoing more invasive treatment developed subsequent complications.^11^, ^18^, ^19^, ^21^ Patient-reported adherence, accessibility and tolerability of the adopted treatment should be considered, as done in four articles.^2^, ^6^, ^12^, ^22^ The change of the adopted treatment in the case of unexpected side effects or recurrence, as reported in seven articles,^2^, ^11^, ^12^, ^18^^,^^19^, ^21^, ^22^ is also important. Finally, the literature reports that pre- (antibiotic prophylaxis) and postoperative antibiotic therapy avoids postoperative complications of invasive dental procedures. In addition, these procedures favor tissue repair in the surgical region, subgingival scraping, and necropulpectomies.^36^, ^37^

## Conclusion

The findings of this review showed that patients with PYCD are more likely to develop osteomyelitis of the jaws, especially after tooth extraction and configure the main risk factor for its establishment. Whenever possible, pre- (antibiotic prophylaxis) and postoperative antibiotic therapy seems to be appropriate in patients with PYCD until complete tissue healing, even in the case of minor procedures, in order to avoid complications such as osteomyelitis and pathological fractures. In addition, more conservative osteomyelitis treatment should be chosen to prevent additional trauma to the jaws.

## Funding

The work was supported by the Department of Dentistry, Oral Pathology Postgraduate Program, Brazil.

## Conflicts of interest

The authors declare no conflicts of interest.
