# Supplementary material for: Osteomyelitis of the jaws in patients with pycnodysostosis: a systematic review
Source: Braz J Otorhinolaryngol. 2021 Jan 29;87(5):620–8. doi: 10.1016/j.bjorl.2020.12.009 (PMC9422419; doi:10.1016/j.bjorl.2020.12.009)
Supplement: Supplementary file 1 [file mmc1.docx]

**BJORL-D-20-00705 – Supplementary Material**

**Supplementary Table 1** Search strategy for each database.

| **Database** | **Search strategy** |
| --- | --- |
| Pubmed: | ((((((((((((((((((((((((((Pycnodysostosis [MeSH Terms]) OR Pycnodysostosis [Title/Abstract]) AND Surgery, Oral[MeSH Terms]) OR Surgery, Oral [Title/Abstract]) OR Maxillofacial Surgery [Title/Abstract]) OR Oral Surgery [Title/Abstract]) OR Exodontics [Title/Abstract]) OR Dentistry, Operative [MeSH Terms]) OR Dentistry, Operative [Title/Abstract]) OR Surgical Procedures, Oral [Title/Abstract]) OR Procedure, Oral Surgical [Title/Abstract]) OR Procedures, Oral Surgical [Title/Abstract]) OR Surgical Procedure, Oral [Title/Abstract]) OR Maxillofacial Procedures [Title/Abstract]) OR Maxillofacial Procedure [Title/Abstract]) OR Procedure, Maxillofacial [Title/Abstract]) OR Procedures, Maxillofacial [Title/Abstract]) OR Tooth extraction [Title/Abstract]) AND Osteomyelitis [MeSH Terms]) OR Osteomyelitis [Title/Abstract]) OR Osteomyelitides [Title/Abstract]) OR Diseases, Infectious Bone [Title/Abstract]) OR Bone Diseases, Infectious [MeSH Terms]) OR Bone Diseases, Infectious [Title/Abstract]) OR Diseases, Infectious Bone [Title/Abstract]) OR Infectious Bone Disease [Title/Abstract]) OR Infectious Bone Diseases [Title/Abstract] |
| Scopus | TITLE-ABS-KEY(Pycnodysostosis) OR TITLE-ABS-KEY(Pyknodysostosis) AND TITLE-ABS-KEY (“Surgery, Oral”) OR TITLE-ABS-KEY (“Maxillofacial Surgery”) OR TITLE-ABS-KEY (“Oral Surgery”) OR TITLE-ABS-KEY (Exodontics) OR TITLE-ABS-KEY (“Dentistry, Operative”) OR TITLE-ABS-KEY (“Surgical Procedures, Oral”) OR TITLE-ABS-KEY (“Procedure, Oral Surgical”) OR TITLE-ABS-KEY (“Procedures, Oral Surgical”) OR TITLE-ABS-KEY (“Surgical Procedure, Oral”) OR TITLE-ABS-KEY (“Maxillofacial Procedures”) OR TITLE-ABS-KEY (“Maxillofacial Procedure”) OR TITLE-ABS-KEY (“Procedure, Maxillofacial”) OR TITLE-ABS-KEY (“Procedures, Maxillofacial”) OR TITLE-ABS-KEY (“Tooth extraction”) AND TITLE-ABS-KEY (Osteomyelitis) OR TITLE-ABS-KEY (Osteomyelitides) OR TITLE-ABS-KEY (“Diseases, Infectious Bone”) OR TITLE-ABS-KEY (“Bone Diseases, Infectious”) OR TITLE-ABS-KEY (“Diseases, Infectious Bone”) OR TITLE-ABS-KEY (“Infectious Bone Disease”) OR TITLE-ABS-KEY (“Infectious Bone Diseases”) |
| Web of science | TITLE: (“Pycnodysostosis”) OR TITLE: (“Pyknodysostosis”) AND TITLE: (“Surgery, Oral”) OR TITLE: (“Maxillofacial Surgery”) OR TITLE: (“Oral Surgery”) OR TITLE: (“Exodontics”) OR TITLE: (“Dentistry, Operative”) OR TITLE: (“Surgical Procedures, Oral”) OR TITLE: (“Procedure, Oral Surgical”) OR TITLE: (“Procedures, Oral Surgical”) OR TITLE: (“Surgical Procedure, Oral”) OR TITLE: (“Maxillofacial Procedures”) OR TITLE: (“Maxillofacial Procedure”) OR TITLE: (“Procedure, Maxillofacial”) OR TITLE: (“Procedures, Maxillofacial”) OR TITLE: (“Tooth extraction”) AND TITLE: (“Osteomyelitis”) OR TITLE: (“Osteomyelitides”) OR TITLE: (“Diseases, Infectious Bone”) OR TITLE: (“Bone Diseases, Infectious”) OR TITLE: (“Diseases, Infectious Bone”) OR TITLE: (“Infectious Bone Disease”) OR TITLE: (“Infectious Bone Diseases”) |
| Lilacs | (mh:(Pycnodysostosis)) OR (mh:(Picnodisostose)) OR (mh:(Picnodisostosis)) OR (tw:(Pyknodysostosis)) AND (mh:(Surgery, Oral)) OR (mh:(Cirurgía Bucal)) OR (mh:(Cirurgia Bucal)) OR (tw:(Maxillofacial Surgery)) OR (tw:(Oral Surgery)) OR (tw:(Exodontics)) OR (mh:(Dentistry, Operative)) OR (mh:(Operatoria Dental)) OR (mh:(Dentística Operatória)) OR (tw:(Surgical Procedures, Oral)) OR (tw:(Procedimientos Quirúrgicos Orales)) OR (tw:(Procedimentos Cirúrgicos Bucais)) OR (tw:(Procedure, Oral Surgical)) OR (tw:(Procedures, Oral Surgical)) OR (tw:(Surgical Procedure, Oral)) OR (tw:(Maxillofacial Procedures)) OR (tw:(Maxillofacial Procedure)) OR (tw:(Procedure, Maxillofacial)) OR (tw:(Procedures, Maxillofacial)) OR (tw:(Tooth extraction)) AND (mh:(Osteomyelitis)) OR (mh:(Osteomielitis)) OR (mh:(Osteomielite)) OR (tw:(Osteomyelitides)) OR (tw:(Diseases, Infectious Bone)) OR (tw:(Bone Diseases, Infectious)) OR (tw:(Enfermedades Óseas Infecciosas)) OR (tw:(Doenças Ósseas Infecciosas)) OR (tw:(Infectious Bone Disease)) OR (tw:(Infectious Bone Diseases)) |
| Cochrane | ID Search Hits |
|  | #1 (“pycnodysostosis”): kw (Word variations have been searched) 1 |
|  | #2 Pyknodysostosis* 0 |
|  | #3 #1 or #2 1 |
|  | #4 (“Surgery, Oral”): kw (Word variations have been searched) 200 |
|  | #5 “Maxillofacial Surgery” 3057 |
|  | #6 “Oral Surgery” 2093 |
|  | #7 Exodontics* 3 |
|  | #8 (“Dentistry, Operative”):kw (Word variations have been searched) 56 |
|  | #9 “Surgical Procedures, Oral” 11 |
|  | #10 “Procedure, Oral Surgical” 0 |
|  | #11 “Procedures, Oral Surgical” 7 |
|  | #12 “Surgical Procedure, Oral” 0 |
|  | #13 “Maxillofacial Procedures” 13 |
|  | #14 “Maxillofacial Procedure” 3 |
|  | #15 “Procedure, Maxillofacial” 0 |
|  | #16 “Procedures, Maxillofacial” 1 |
|  | #17 “Tooth extraction” 2413 |
|  | #18 #4 or #5 or #6 or #7 or #8 or #9 or #10 or #11 or #12 or #13 or #14 or #15 or #16 or #17 6463 |
|  | #19 (“osteomyelitis”):kw (Word variations have been searched) 331 |
|  | #20 Osteomyelitides* 0 |
|  | #21 “Diseases, Infectious Bone” 1 |
|  | #22 (“Bone Diseases, Infectious”):kw (Word variations have been searched) 8 |
|  | #23 “Diseases, Infectious Bone” 1 |
|  | #24 “Infectious Bone Disease” 1 |
|  | #25 “Infectious Bone Diseases” 1 |
|  | #26 #19 or #20 or #21 or #22 or #23 or #24 or #25 339 |
|  | #27 #3 and #18 and #26 0 |

**Supplementary Table 2** Etiology, clinical and radiographic characteristics of the 27 cases of osteomyelitis in patients with pycnodysostosis.

| **Authors** | **Nº of cases** | **Etiology and causal factors** | | | | **Promote factor** | **Clinical and radiographic characteristics** | | | **Treatment** | **Antibiotics regimen** | **Follow-up** |
| --- | --- | --- | --- | --- | --- | --- | --- | --- | --- | --- | --- | --- |
|  |  | **Average age (years)** | **Gender**  **M/F** | **Family histoy** | **Medical history** |  | **Localization** | **Simptomatology** | **Radiographic features** |  |  |  |
| Emami-Ahari et al., 1969[15] | 1 | 37 | 1/0 | Consanguineous marriage | Long bone fractures | Exodontics | Lower jaw | Purulent secretion  fistula | Osteosclerosis,  Fracture and bone sequestration | Sequestrectomy and curettage | NI | 8 months |
| Yamada et al., 1973[30] | 2 | 27 | 0/1 | No family member with the disease | Tibial Fractures | Exodontics | Lower jaw | NI | Presence of bone sequestration | Antibiotic therapy and curettage | NI | NI |
|  |  | 46 | 1/0 | Consanguineous marriage | Femoral Fracture | Exodontics | Lower jaw | Absent | Presence of bone sequestration | Sequestrectomy | NI | NI |
| Green & Rowe, 1976[21] | 1 | 42 | 1/0 | NI | Tibial and rib fractures | Exodontics | Lower jaw | Purulent Secretion | Osteosclerosis and fracture | Antibiotic therapy and reconstruction with titanium prosthesis | Picillin and flucloxacillin | 9 months |
| Zachariades & Koundouris, 1984[18] | 2 | 37 | 1/0 | 1 brother | Femoral and elbow fracture | Fracture of the jaw | Lower jaw | Purulent Secretion | Radiolucency with ill-defined edges | Curettage, resection and antibiotics | NI | 24 months |
|  |  | 35 | 1/0 | 1 brother | Femoral and elbow fracture | Exodontics | Upper jaw | Purulent Secretion | Radiolucency with ill-defined edges | Curettage and antibiotics | Bacterial culture and antibiotic sensitivity testing showed organisms sensitive to carbenicillin and ampicillin. | Death 2 days after surgery procedure |
| Van merkesteyn et al., 1987[19] | 3 | 41 | 0/1 | NI | NI | Exodontics and fracture of the jaw | Lower and upper jaw | Pain and fistula | Osteolysis | Sequestrectomy and antibiotics | Penicillin G i.v., 16 million units daily for 5 days | 19 months |
|  |  | 29 | 0/1 | NI | NI | Exodontics | Upper jaw | Pain, swelling and fistula | Osteolysis and bone sequestration | Sequestrectomy and antibiotics | 12 million units of penicillin G i.v., for 7 days, followed by feneticilline, orally, 2500 mg a day for 21 days. | 7 months |
|  |  | 23 | 0/1 | NI | NI | Exodontics | Lower jaw | Swelling | Bone sequestration | Sequestrectomy and antibiotics | 4.8 million units of bicilline i.m. | Death with 5 days |
| Mills & Johnston, 1988[27] | 1 | 40 | 1/0 | 1 brother | Long bone fracture. | Exodontics | Lower jaw | NI | Osteosclerosis | Antibiotic therapy | NI | NI |
| Iwu, 1991[23] | 1 | 25 | 1/0 | NI | NI | Fracture of the jaw | Lower jaw | Swelling and fistula | Osteosclerosis and bone sequestration | Sequestrectomy and antibiotic therapy | Oral lincomycin, 500 mg four times daily for 10 days. | NI |
| Muto et al., 1991[28] | 1 | 55 | 0/1 | NI | Fracture of long bones and shoulder blade | Exodontics | Lower jaw | Pain | Osteosclerosis and bone sequestration | NI | NI | 168 months |
| Schmitz et al., 1996[22] | 1 | 45 | 1/0 | No family member with the disease | Hip fractures, tibia  and ribs | Exodontics | Lower jaw | Pain | Osteosclerosis and osteolysis. | Resection with reconstruction and antibiotic therapy | Clindamycin, and intravenous timentin | 24 months. |
| Alibhai et al., 1999[20] | 1 | 30 | 1/0 | 6 brothers | Long bone fracture | Exodontics | Lower jaw | Pain, trismus and swelling | Hypercementosis, osteosclerosis. | Sequestrectomy and antibiotic therapy | Penicillins (1.2 mU i.m. once a day), ampicillin (500 mg thrice daily) given at different times for 5 days | NI |
| Bathi & Masur, 2000[29] | 1 | 38 | 1/0 | No family member with the disease | Hydrocephalus, elbow, knee and hip fractures | Periodontal disease | Upper jaw | Pain and swelling | Osteosclerosis, hypercementosis | Antibiotic therapy, exodontics and curettage | NI | NI |
| Kirita et al., 2001[11] | 1 | 21 | 1/0 | No family member with the disease | Long bone fractures and hydrocephalus | Periodontal disease | Lower jaw | Swelling and Purulent Secretion | Bone sequestration and osteolysis. | Sequestrectomy, antibiotics and reconstruction | NI | 17 months |
| Kato et al., 2005[13] | 1 | 48 | 1/0 | Consanguineous marriage | Repeated fractures of the lower extremities | Exodontics | Lower jaw | Swelling | Fracture and osteolysis | Sequestrectomy | NI | 25 months |
| Dimitrakopoulos et al., 2007[16] | 1 | 50 | 0/1 | No family member with the disease | NI | Caries and periodontal diseases | Upper jaw | Pain and swelling | Bone sequestration and osteolysis. | Sequestrectomy and antibiotic therapy | Penicillin + clavulanic acid, and fusidic acid intravenously | 6 months |
| Frota et al., 2010[14] | 1 | 45 | 0/1 | 2 brothers | Clavicle fracture | Exodontics | Lower jaw | Oedema and erythema associated with fistula | Osteosclerosis and osteolysis. | Sequestrectomy with reconstruction and antibiotic therapy. | Bacterial culture and antibiogram testing showed organisms sensitive to clindamycin, initiated 2 weeks before the surgical procedure and extended for 4 months in accordance with the treatment proposed for chronic osteomyelitis | 36 months |
| Rohit et al., 2015[5] | 2 | 40 | 0/1 | Consanguineous marriage, brother and paternal aunt affected. | NI | Carie and impacted tooth | Lower jaw | Swelling and pus | Bone sequestration | Exodontics, antibiotic therapy and curettage | NI | NI |
|  |  | 36 | 1/0 | NI | NI | Pathological fracture | Lower jaw | Swelling and purulent secretion | Bone sequestration | Exodontics, antibiotic therapy and curettage | NI | NI |
| Kamat et al., 2015[2] | 1 | 36 | 0/1 | NI | Lower limb fractures | Exodontics | Lower jaw | Pain and purulent secretion | Bone sequestration | Antibiotic Therapy | Systemic and local antibiotic therapy for 3 years. Bacterial culture and antibiogram test showed microorganisms sensitive to gentamycin, imipenem and azithromycin. Gentamicin sulfate 80mg/2 ml thrice daily was used in the form of irrigations through the extra-oral sinus tract and intra-oral mucosal opening | 36 months |
| Fénelon et al., 2015[12] | 2 | 50 | 1/0 | NI | Long bone fractures | Caries | Upper and lower jaw | Pain | Bone sequestration and  Osteosclerosis. | Sequestrectomy,  Curettage, exodontics and antibiotic therapy | Amoxicillin 1g, twice daily for 15 days | 12 months |
|  |  | 53 | 1/0 | NI | Long bone fractures | Periodontal disease and pathological fracture | Lower jaw | Pain | Hypercementosis and bone sequestration | Antibiotic therapy | Amoxicillin + clavulanic acid for 7 days | 12 months |
| Dhameliya et al., 2017[17] | 1 | 25 | 0/1 | No family member with the disease | Femoral Fractures | Caries and pathological fracture | Lower jaw | Swelling and purulent secretion | Fracture, osteosclerosis and bone sequestration | Sequestrectomy | NI | NI |
| Oliveira et al., 2018[6] | 1 | 30 | 0/1 | NI | Clinical fractures | Exodontics | Lower jaw | Pain | Bone sequestration | Antibiotic therapy, sequestrectomy and closure with free buccal fat pad | Clindamycin 300 mg, four times daily for 14 days | 24 months |

NI, Not informed.
